# Crystallization-Based Modification of Ammonium Perchlorate Heat Release

**DOI:** 10.1021/acs.cgd.4c00769

**Published:** 2024-09-06

**Authors:** Natalie Smith-Papin, Cynthia Do, Meagan Phister, Gaurav Giri, Joseph Kalman

**Affiliations:** † Department of Chemical Engineering, University of Virginia, Charlottesville, Virginia 22904, United States; ‡ Department of Mechanical and Aerospace Engineering, California State University Long Beach, Long Beach, California 90840, United States

## Abstract

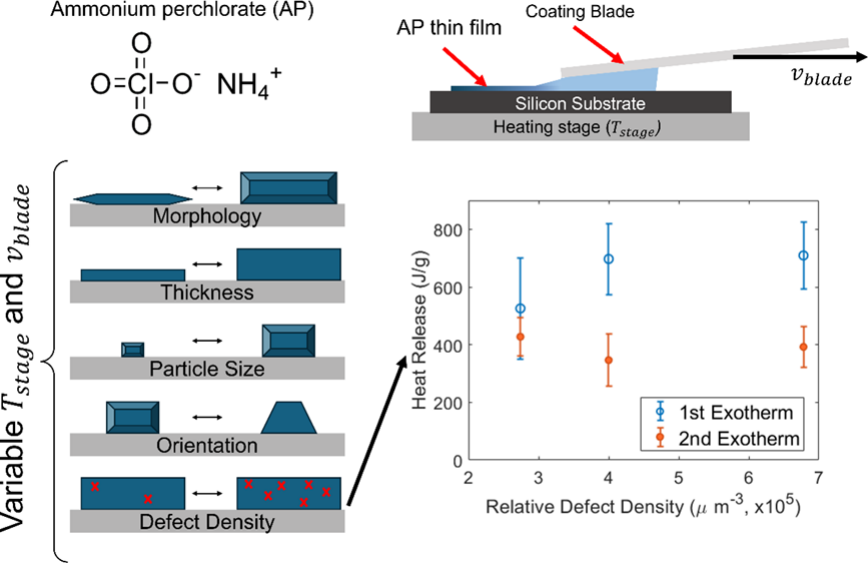

Composite propellants use the decomposition of crystalline oxidizers, such as ammonium perchlorate (AP), to produce oxidizing species that can combust with fuels. Controlled crystal microstructure must be leveraged to tailor reactivity to minimize the use of exotic energetic materials. This work uses meniscus-guided coating (MGC) to fabricate films of AP with a high degree of control over the AP crystal microstructure. Exploring a wide range of crystallization parameters resulted in film thickness ranging from 200 nm to 14 μm, particle size ranging from 18 to 110 μm, variable preferential orientation with respect to the substrate, and relative defect density ranging from 2.74 × 10^5^ to 6.78 × 10^5^ μm^–3^. Increasing coating blade speed and substrate temperature within the MGC process shifts the preferential orientation of the AP crystals from predominantly exhibiting (002) and (210) crystal planes parallel to the substrate to (200)/(011) crystal planes parallel to the substrate. This shift in orientation is accompanied by an increase in defect density, which is shown to increase the heat release from the low-temperature decomposition regime and decrease the heat release from the high temperature regime. These results demonstrate the ability to use recrystallization, defect density control, and orientation control to tune the heat release profiles of energetic materials to augment propellant performance.

## Abstract 2

Ammonium perchlorate thin films were fabricated using meniscus-guided coating, with control over the crystal microstructure. Morphology, film thickness, particle size, preferred crystallographic orientation, and relative defect density vary with crystallization temperature and coating blade speed. The results demonstrate ability to use crystallization control to tune heat release properties of energetic materials to enhance propellant performance.

## Introduction

Ammonium perchlorate (AP) is commonly used as an oxidizer for solid rocket propellant due to its excess oxygen balance and long-term storability.^1−5^ The thrust produced is directly related to the burning rate of the propellant, and thus, formulating a mission-specific propellant requires the ability to design the propellant to meet a specific burning rate. One method commonly employed to control the burning rate is to control the AP particle size(s) within the propellant formulation. The particle size dictates the combustion mechanism by the location of heat release (e.g., flame structure),^6^ which controls the conductive heat feedback to the propellant surface.^7,8^ The addition of burning rate modifiers is commonly used to augment the burning rate as evident by the nearly 10x increase in published works over the last two decades.^9^ Additional efforts have been made toward cocrystallization to adjust performance, environmental aspects, insensitive munitions, etc.^1,10^

AP decomposition has long been hypothesized to be initiated by charge transport between the ammonium and perchlorate ions within the condensed phase.^11,12^ Recent work has proposed a new mechanism where the perchlorate ion decomposes prior proton transfer.^13^ Regardless, proton transfer is still believed to be a critical step in the AP decomposition. Burning rate modifiers (BRMs) were used to accelerate this decomposition process. However, there is evidence that BRMs impact proton and/or charge transfer, catalyze gas phase reactions, and/or alter the system thermodynamically.^9^ In addition, ionic BRMs may also disrupt the AP crystal lattice resulting in an increased amount of undesired decomposition in isothermal conditions^14^ or gas production prior to exothermic reactions.^15^ These results emphasize the connection between the AP crystal structure and the decomposition process^11,16^ and reactivity.^17^ Isothermal decomposition of cubic AP indicated that the level of compression prior to heating affected the rate and extent of decomposition. These results suggest that the defects present in the crystal affect the decomposition process.^18^.

Crystalline defects have a locally high surface energy due to the nature of the broken bonds at the defect site. Computational work^19^ determined the ordering of the most energetically stable crystalline planes of AP that has displayed some agreement with experimental work.^20^ Changes to the solvent were shown to alter the surface energy of the planes.^21^ Recrystallized AP studies^22,23^ using ethylene glycol showed habit modification and an increased extent of the (210) plane, which altered the decomposition and combustion rates of AP and AP propellants, respectively. Low-temperature decomposition is well-known to have highly anisotropic dependencies on reactivity.^16^ This body of research indicates that AP decomposition is controlled by the crystal habit and defects within the crystal.

Despite these and other studies recrystallizing AP, there is an absence of detailed research in which the AP crystallization process is highly controlled. Recent work with meniscus-guided coating (MGC) has been able to fabricate thin films of energetic materials, semiconducting molecules, and pharmaceutical crystals by controlling solvent evaporation rates and inducing shear.^24−28^ This approach provides an additional level of control compared to crash precipitation, since the blade speed and substrate temperature dictate the evaporation and deposition rates, influencing the crystallization pathway. In this work, MGC coating was used to recrystallize the AP into a thin film. These recrystallized AP samples were fabricated with different processing conditions, by varying the MGC speed (0 – 0.3 mm/s), substrate temperature (20 – 80 °C), concentration of AP (15 – 30 mg), and addition of antisolvent (ethyl acetate). Samples were analyzed with X-ray diffraction (XRD), microscopy, profilometry, computed tomography (CT), and differential scanning calorimetry/thermogravimetric analysis to link the crystal microstructure to the thermal decomposition kinetics. By tuning the MGC parameters, films with thickness ranging from 200 nm to 14 μm, particle size ranging from 18 to 110 μm, and thin film preferential orientation of (002), (210), and (200)/(011) can be obtained. Further, higher defect density, observed at higher fabrication temperatures, led to a change in decomposition kinetics, resulting in a higher energy output at lower temperature for the subset of crystals tested. Thus, controlled recrystallization processes based on the knowledge from MGC can be utilized to tune the performance of AP. In addition, the knowledge of defect density in AP can be used to obtain structure–property relationships for AP utilization.

## Materials and Methods

### Materials

Ammonium perchlorate (AP) was obtained from Firefox Enterprises. Methanol (Alfa Aesar, 99.8%), ethyl acetate (Fisher Scientific, 99.9%), toluene (Sigma Aldrich, 99.5%), and trichloro(octadecyl)silane (OTS, Sigma Aldrich, >90%) were used as obtained. Silicon wafers with 500 μm thickness and a single-side polished surface finish were purchased from University Wafer.

### MGC Blade Functionalization

A silicon wafer was cut into a 4 cm × 3 cm rectangle with the flat, linear side intended as a contact edge for the MGC blade. The blade was washed with toluene, acetone, DI water, and isopropyl alcohol (IPA). Compressed air was used to dry the blade. The blade was treated with UV/ozone treatment for 20 min to create a clean, hydrophilic surface before being placed in a large crystallization dish with 50 mL of toluene and 200 μL of OTS. The dish was covered, heated at 50 °C, and stirred for 20 h to allow the OTS to chemisorb onto the surface of the silicon wafer. After removal and drying, the blade was thermally annealed at 90 °C for 1 hour. Finally, the blade was sonicated in acetone for 10 min to remove any physisorbed material from the surface. DI water contact angle was observed to ensure that a hydrophobic surface remained after the OTS functionalization.

### MGC Equipment

An in-house MGC equipment was fabricated using an aluminum block (designed in-house, machined by Protolabs), high temperature heating cartridges (McMaster-Carr), and J-type thermocouples (McMaster-Carr). A proportional integral derivative (PID) controller (Omega Engineering Inc.) was used to heat aluminum base and ensures that temperature is maintained at set point. A custom-made blade holder with angle/yaw (OptoSigma) and height (Edmunds Optics) micromanipulators was controlled by using a motorized liner driver (Zaber Technology) to move the coating blade translationally at a fixed speed. A vacuum connection in the aluminum heating block was used to hold the sample substrate in place during the coating process.

### Substrate Preparation

Thin films of AP were coated onto silicon wafer substrates. Silicon wafers were cut into roughly 1.5 cm × 1.5 cm squares and then washed with toluene, acetone, DI water, and isopropyl alcohol. Compressed air was used to dry the substrates before exposing them to UV/ozone treatment for 20 min to create a hydrophilic surface and improve solvent wetting during the coating process. After removal from the UV/ozone treatment, the substrate was placed on the MGC heating stage and held in place via a vacuum connection prior to coating.

### AP Solution Preparation

Two concentrations of AP with solvent (methanol) and solvent-antisolvent (methanol/ethyl acetate) systems were studied. The solvent system solution was prepared at a concentration of 30 mg of AP and 2 mL of MeOH. The solvent–antisolvent system solutions were prepared at concentrations of 15 mg of AP and 1 mL of MeOH and 1 mL of EtOAc and 30 mg of AP and 1 mL of MeOH and 1 mL of EtOAc. Solutions were stirred on a stir plate before coating onto silicon substrates.

### MGC Technique

Thin films of AP were crystallized using the in-house MGC equipment described above. The temperature control was turned off for room temperature (∼20 °C) and set to 40 °C to study the impact of increasing temperature on the evaporation rate and film formation. The translational coating speed was set to 0.01, 0.05, 0.15, and 0.3 mm/s. After the substrate was thermally equilibrated with the heating stage, 30 μL of AP solution was pipetted between the coating blade and the substrate before the blade began to translate at the fixed speed designated. Once the blade began to move, an evaporation front developed, allowing the solvent(s) to leave and the solute to deposit onto the silicon substrate as a thin film. Three films were made for each MGC condition.

### Dropcasting Technique

The aluminum block from the MGC technique was utilized as a hot plate for dropcast samples. The MGC blade was moved away from the stage, and a substrate was placed on the aluminum block. The heating elements were turned off for the room temperature (∼20 °C) samples and turned on to 40 and 80 °C for the higher temperature samples. Once the silicon wafer substrate was thermally equilibrated, 30 μL of solution was pipetted onto the substrate, the solvent(s) evaporated, and a thin film of AP formed. Three films were made for each solution and temperature combination.

### Polarized Optical Microscopy

A Zeiss Axio Imager A.1 optical microscope (Carl Zeiss AG) in tandem with a Zeiss Axiocam 503 Color camera (Carl Zeiss AG) was utilized to image the thin film samples. Bright-field images were collected for all films. Additionally, polarized images were collected by inserting two polarizers oriented orthogonally into the light path to produce linearly polarized light. This setup allows for the visualization of alignment and isotropy within the thin films.

### Particle Size Analysis

ImageJ software was utilized with bright-field images to determine the average particle size and coverage for the films. For particle size measurements, the scale was set in the software; over twenty crystals were measured along the dimension associated with the coating direction, and those values were averaged to determine the average particle size for each MGC condition.

### Profilometer

A Bruker DektakXT Stylus Profiler was utilized to measure the film thickness with a scan speed of 10 μm/s and a stylus force of 1 mg. A razor blade was used to remove a channel of material from the middle of the film, exposing the silicon substrate. The stylus then measured the height of several hundred micrometers of the film prior to dropping off the ledge of the channel and measuring the relative height difference between the top of the film and the top of the substrate. The average step height was measured for each film.

### Raman Spectroscopy

A Renshaw inVia Confocal Raman Microscope (spatial resolution < 2 μm) with the following settings, 514 nm laser and 1800 L/mm grating, 50% power, 5 s exposure, and 5 accumulations, was used to measure the Raman shift (100–3500 cm ^–1^) for each of the films.

### X-Ray Diffraction

A Malvern PANalytical Empyrean diffractometer with Bragg-Bretano scanning geometry was used to acquire the diffraction patterns for each of the films. X-rays were generated via Cu K-α radiation and accelerated by a 45 kV voltage and 40 mA beam current. Data were processed using Spectragryph 1.2.16.1, and background subtraction was performed prior to peak intensity and full width-half-maximum (fwhm) analysis for preferential orientation and coherence length results.

### Differential Scanning Calorimetry/Thermogravimetric Analysis (DSC/TGA)

Thermal analysis of the samples was performed to determine the thermal decomposition behavior of the recrystallized material. A simultaneous differential scanning calorimetry (DSC)–thermogravimetric analysis (TGA) system (TA Instruments SDT 650) was used to analyze the samples. In each experimental run, about 10–15 mg of the sample was placed in open alumina pans (90 μL of alumina, P/N: 960070.901). Experiments were carried out in an argon (industrial grade) atmosphere with a flow rate of 100 mL/min at a heating rate of 30 °C/min and temperature range of 80–600 °C. Once the thermal analysis was completed, the information was processed through Trios and MATLAB. Trios was used to find the enthalpy (energy release of the system) from the area under the peaks from the DSC data. MATLAB scripts were written to find the temperature of the beginning of the mass loss (taken to be after 1 wt % of initial mass was lost) and rapid mass loss of the data. The maximum rate of mass loss was found by finding the slope of the first derivative of the mass loss data and identifying the inflection points. The inflection points describe the change in the thermal decomposition rate, which was used to find the maximum rate of mass loss.^15^

### Computed Tomography (CT)

Samples were measured at Advanced Photon Source beamline 7-BM-B for CT scan characterization. A custom aluminum mount held the silicon wafer with deposited AP in place. The camera detector (FLIR Oryx ORX-10G-310S9M) with a 10× microscope objective recorded data at a rate of 18 frames/s with an exposure time of 0.100 s. Transmitted X-ray photons were incident on a 25 μm thick LuAG:Ce scintillator positioned 75 mm from the sample center. The beamline source current used was 102 mA. The effective voxel size of the CT scan images was 0.7 μm per voxel. The reconstructed images from the CT scan measurements were imported to image processing software (Dragonfly by Comet Technologies). Dragonfly was utilized to produce images in both 2D and 3D by layering the sliced images vertically to produce a visualization of the recrystallized films. From these images, each individual particle was identified, and the volume particle measurements were measured. The Deep Learning Tool from Dragonfly was used to find the volume measurements. A raw sample slice was marked through user input to identify the crystals on the recrystallized film and was used as a reference for the image processing program to find all of the crystal measurements.

## Results

**Figure 1:**
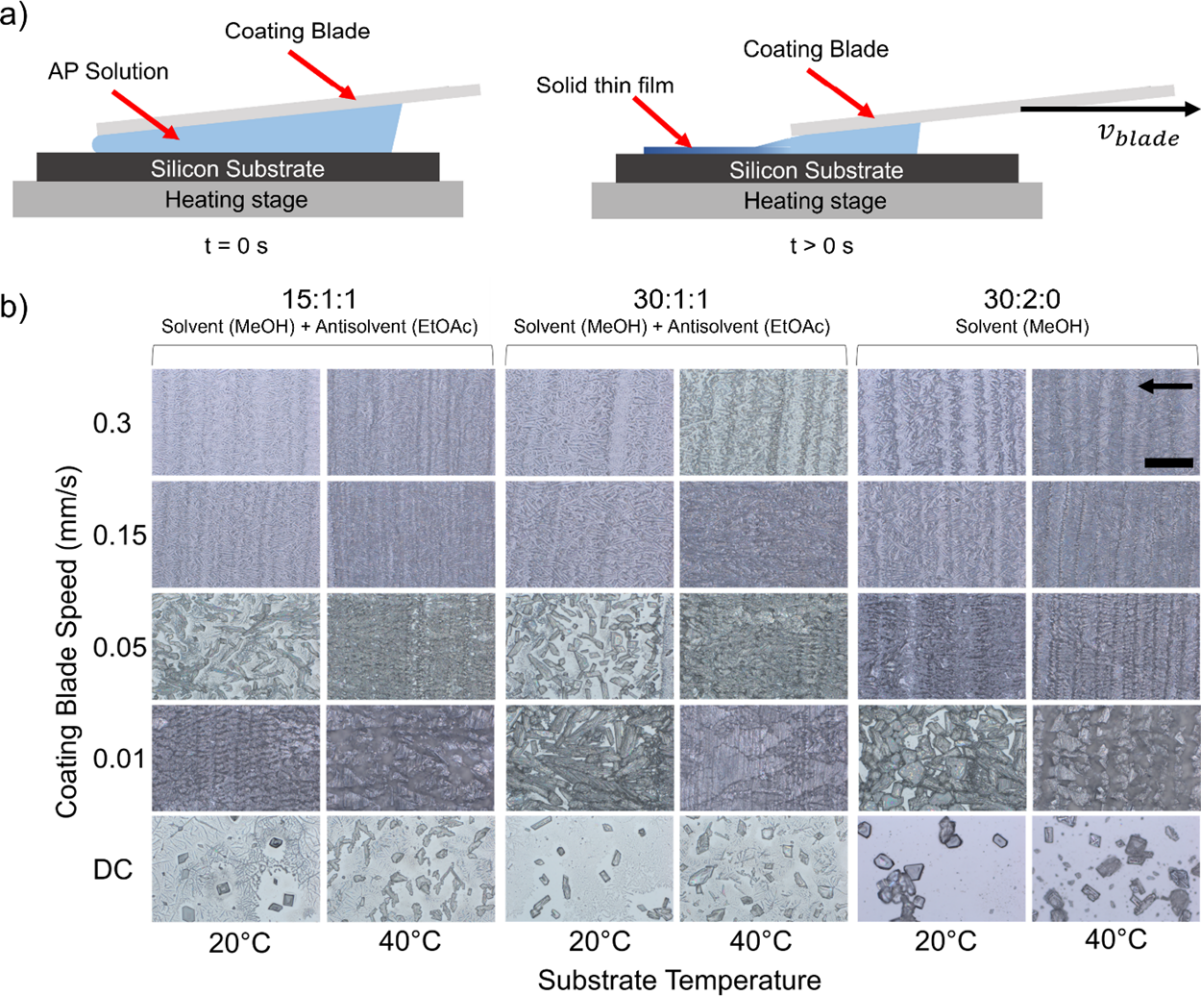
(a) Schematic depicting the MGC technique. Solution containing AP is sandwiched between the substrate and the coating blade. As the blade then moves, the solvent evaporates, leaving a thin film of the solid AP. (b) Optical images of all samples made within the parameter space chosen. Coating blade speed increases along the *y*-axis from dropcast (DC) to 0.3 mm/s and temperature increases along the *x*-axis from 20 to 40 °C for three different solvent/antisolvent concentrations (15:1:1, 30:1:1, 30:2:0, where *x*:*y*:*z* indicates mg AP: mL MeOH: mL EtOAc). Scale bar (100 μm) in the top right image is the same for all images, and arrow in the top right image indicates the direction of the coating blade.

In this study, MGC was utilized to induce evaporative crystallization of AP by injecting a solution containing AP between a coating blade and a heated substrate (Figure 1a). The coating blade translates linearly at a designated speed, opening a meniscus where the solvent evaporates. As the solution reaches supersaturation, AP crystallizes onto the substrate as a thin film. For this work, both solvent and solvent–antisolvent systems were used to determine how incorporating an antisolvent changes the dynamics during evaporative crystallization via MGC. Temperatures of 20 and 40 °C were chosen, as they are below the boiling point of both solvent (MeOH bp 64.7 °C) and antisolvent (EtOAc bp 77.1 °C) and do not result in immediate evaporation prior to blade coating. Dropcast samples, as well as samples with coating blade speeds of 0.01 mm/s. 0.05, 0.15, and 0.3 mm/s, were chosen to explore a wide parameter space. By varying the solute concentration, substrate temperature, coating blade speed, and presence of antisolvent during crystallization, we expect to observe a variety of particle sizes, morphologies, film thickness, and preferred crystallographic orientations in AP thin films, which would ultimately influence the decomposition behavior of the AP.

After AP films were crystallized by using dropcast and MGC techniques, optical microscopy was utilized to characterize the film morphology and particle size distributions. Optical images for various MGC conditions show unique morphology relative to dropcast (DC) samples (Figures 1b and S1). Films crystallized at faster coating speeds (0.3 mm/s) exhibit feather-like, elongated morphology, while films crystallized at slower coating speeds (≤0.05 mm/s) exhibit a more isotropic morphology. With faster coating speeds, the meniscus spans a longer distance and allows for a larger area where evaporation occurs, producing an overall faster evaporation of the solvent.^25,29,30^ Typically, faster evaporation leads to a shorter time scale for supersaturation to occur and results in a nucleation-dominated regime where more particles are observed per unit area, but particles are relatively smaller. Most films crystallized using MGC from all solvent and solvent–antisolvent systems were visually observed to have crystalline particles aligned in the coating direction (indicated by the black arrow in the top right image of Figure 1b). Further, SEM micrographs were collected for three sample conditions (30:1:1, 20 °C, DC; 30:1:1, 20 °C, 0.05 mm/s; and 30:1:1, 20 °C, 0.3 mm/s) representative of different morphological regimes to demonstrate agreement between optical microscopy and SEM (Figure S2).

**Figure 2:**
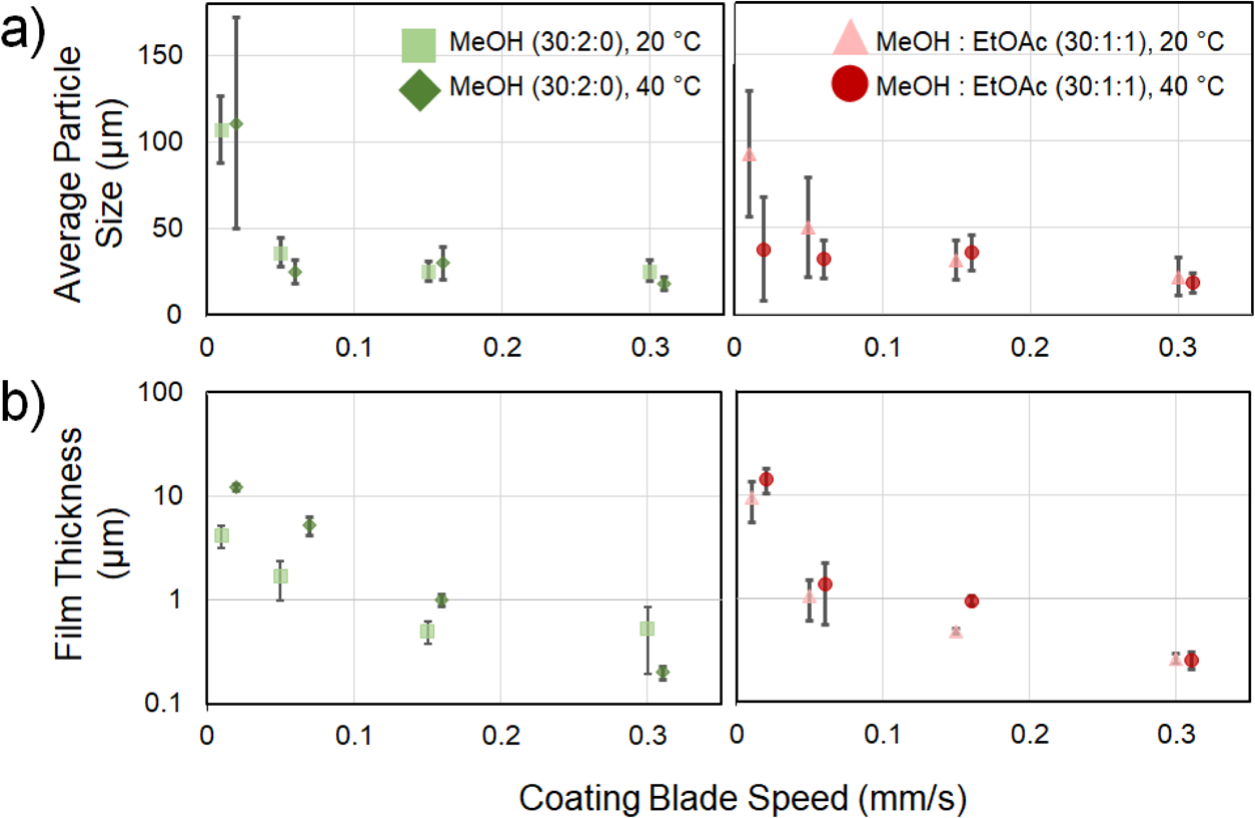
(a) Average crystal size and (b) average film thickness for sample solution sheared at 20 and 40 °C, and using only MeOH as solvent, or MeOH: EtOAc 1:1 v/v.

Film characteristics, including particle size distribution and film thickness, can be controllably tuned by altering the MGC conditions during the crystallization process.^24,25^ The evaporation and deposition rates during MGC are dependent on the solvent/antisolvent type, solute concentration, substrate temperature, and coating blade speed. The crystal size distribution was measured by using the optical images in ImageJ software (Figure 2a). Measurements of 20 crystals were taken along the MGC direction, and the crystal size decreases with increasing coating blade speed. For dropcast samples, crystals with an average size of up to 110 μm were observed for samples with 30 mg AP: 2 mL MeOH: 0 mL EtOAc, substrate temperature of 40 °C. Within the MGC parameter space, average crystal size ranged from 18.1 μm (for samples with 30 mg AP: 2 mL MeOH: 0 mL EtOAc, substrate temperature of 40 °C, and blade speed of 0.3 mm/s) to 50.2 μm (for samples with 30 mg AP: 1 mL MeOH: 1 mL EtOAc, substrate temperature of 20 °C, and blade speed of 0.5 mm/s). Substrate temperature does not seem to significantly influence the crystal size over the parameter space chosen.

Film thickness was characterized, and measurements indicate tunable thickness between 200 nm and 14 μm (Figure 2b). Film thickness decreases with increasing coating blade speed, indicating that film formation occurs in the evaporative regime.^31^ Thickness also increases with increasing temperature over the coating speeds measured as increasing temperature drives faster evaporation from the meniscus and more material deposition per unit area over the substrate surface. The MGC technique provides a high degree of control over the particle size and film thickness, ultimately providing a platform for controlling the crystal characteristics and the resultant material properties.

**Figure 3:**
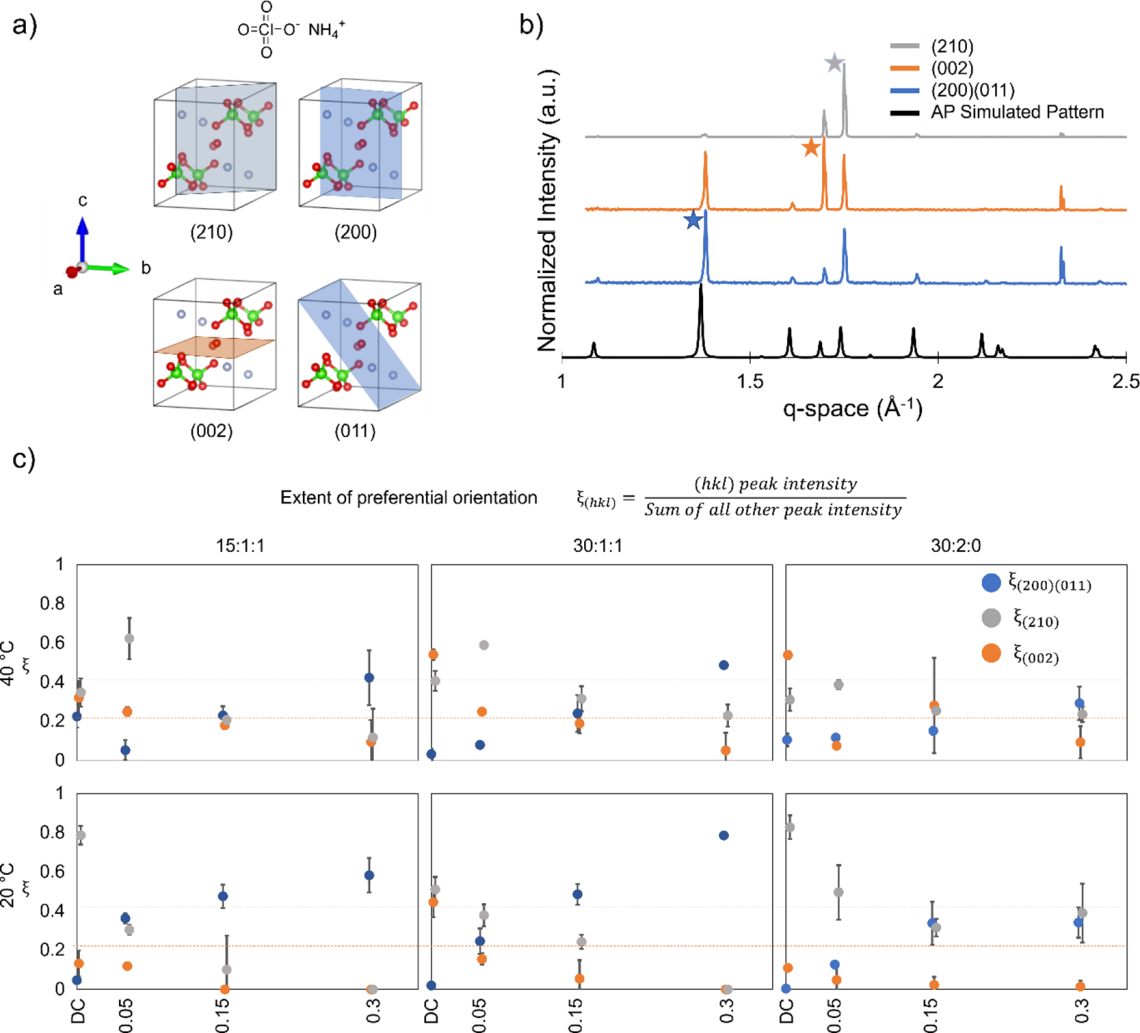
(a) AP chemical structure, unit cell, and schematic highlighting different crystallographic planes within the unit cell (VESTA software used for unit cell visualization^33^); (b) representative XRD spectra for each preferential orientation observed, with the star showing the highest intensity peak variation between different thin films; and (c) extent of preferential orientation of the (200)(011), (210), and (002) crystallographic planes. Dashed lines represent the expected (210)/(002) ratio from scattering from an isotropic powder.

AP exhibits an orthorhombic crystal structure where *a* = 9.20 Å, *b* = 5.82 Å, and *c* = 7.45 Å.^32^ Crystallographic planes within the unit cell of AP (Figure 3a) have unique surface energies. These surface energies have been computed for the major crystallographic planes that are observed as interfaces in experiments.^21^ Each crystallographic plane has an associated peak in the XRD spectra for AP, and higher intensity ratios beyond the isotropic peak intensity indicate a higher degree of preferential orientation of that crystallographic plane with respect to the substrate.

XRD was utilized to characterize the preferential orientation and coherence length of the crystals within the films. Three different crystal planes were observed to be the dominant orientation within different regions of the parameter space studied, as characterized by an increased intensity of the X-ray scattering from that crystal plane. The peak at reciprocal space (*q*) = 1.37 Å^–1^ is associated with the (200) and (011) planes, the peak at 1.69 Å^–1^ is associated with the (002) plane, and the peak at 1.74 Å^–1^ is associated with the (210) plane. The (200) and (011) reflections occur at the same q-space value, and deconvoluting the contribution of each of these peaks was not possible with the resolution of the X-ray diffraction instrument. However, the most intense peak within each spectrum indicates that the associated crystallographic plane is preferentially oriented parallel to the substrate. Representative spectra for each preferential orientation are presented in Figure 3b.

The extent of preferential orientation, ξ_(*hkl*)_, for the crystallographic plane (*hkl*) is defined as ξ_(*hkl*)_ = [(*hkl*) peak intensity]/[sum of all peak intensities], where the peaks for AP according to the American Society for Testing and Materials (ASTM) are listed in Table S1.^22^ ξ_(200)(011)_, ξ_(002)_, and ξ_(210)_ are calculated for each sample condition (Figure 3c). Each of these orientations is preferred within different regions of the parameter space. Samples dropcast with 30:2:0 and 30:1:1 concentration ratios as defined earlier, and at 40 °C, preferentially exhibit the (002) orientation (Figure 3c). Other samples dropcast or coated with speeds > 0.1 mm/s and at 40 °C exhibit preferential (210) orientation. For all solvent systems and concentrations, we consistently observe an increase in (200)/(011) orientation and a decrease in (210) orientation as coating blade speed increases. Relating this finding to film thickness measurements, we observe that thinner films (<0.5 μm) preferentially exhibit (200)/(011) orientation and thicker films (>0.5 μm) preferentially exhibit (210) or (002) orientations. Antisolvent addition in crystallization decreases the relative intensity of the (210) crystal plane and increases the relative intensity of the (200)/(011) planes.

Based on previous work, the relative surface energy of AP crystallographic planes is calculated as (001) < (210) < (101) < (100) < (011).^21^ As the substrate temperature increases, the evaporation rate increases, and crystallization occurs more rapidly, providing less control over the crystallization process. In these scenarios, we believe that the crystal plane with the minimum surface energy becomes more difficult to attain during rapid crystallization as the dominant interface and higher energy surfaces have an increased probability of being stabilized. Similarly, increasing the coating blade speed provides a larger area where evaporation can occur, and inclusion of an antisolvent causes the system to reach supersaturation more rapidly. Both of these effects ultimately result in more rapid crystallization and less time for the bulk equilibrium crystal morphology to be obtained. Preferentially growing different crystallographic orientations can provide a handle for controlling burn rate and sensitivity^22,23^ as well as provide a platform for experimentally studying decomposition kinetics along different crystallographic axes.

Further, XRD of these samples can be used to obtain the coherence length of the crystallites. Smaller crystal coherence lengths indicate an increase in the number of defects. Coherence lengths were calculated using the following equation: CL_(*hkl*)_ = [2π*K*]/[Δ*q*_(*hkl*)_], where *K* is the Scherrer constant (chosen as 0.94) and Δ*q*_(hkl)_ is the full width at half max (fwhm) of the (*hkl*) plane peak.^34^ The strongest intensity peak, indicative of the preferential orientation, was chosen for the coherence length calculations.

**Figure 4:**
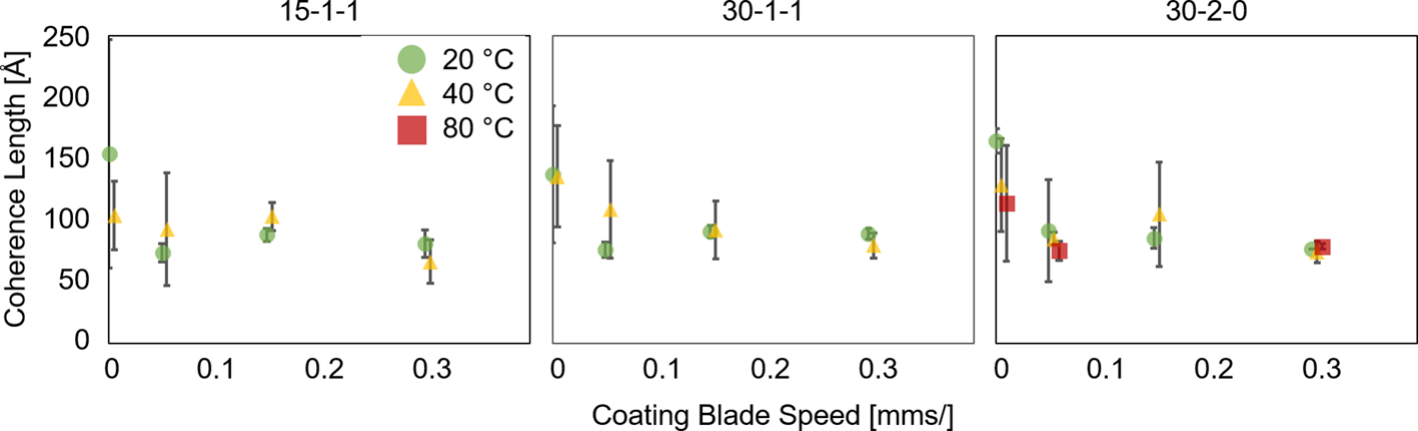
Coherence length of the XRD peaks calculated for 15-1-1 (left), 30-1-1 (middle), and 30-1-0 (right) concentrations at various coating blade speeds and temperatures. Sample size = 3.

Across all recrystallized samples, the coherence length appears to stay constant within experimental error as the coating blade speed increases (Figure 4). We expect that with faster coating speeds, a larger area for evaporation develops along the meniscus and faster evaporation induces more rapid nucleation and growth, ultimately leading to a higher degree of defects in the resultant crystals. Interestingly, the coherence length does not appear to be influenced substantially by changes in the crystallization temperature. We conclude that the crystallization occurs in a kinetic regime in all cases where the molecular attachment to the growing crystal results in a high base defect rate.

To relate the influence of defects in the crystal structure to the resulting decomposition behavior, we selected a smaller subset of dropcast samples (Table 1) for two main reasons. First, larger crystals produced during dropcast allow for more accurate volume measurements using CT. Second, dropcast samples are assumed to have a more isotropic distribution of defects relative to coated samples, where defect density may be enhanced at the air or substrate interface. Samples were dropcast at various temperatures (20, 40, and 80 °C) to determine the influence of processing temperature on microstructure defects. This set of conditions is not intended to comprehensively evaluate the effect of all parameters but to demonstrate the ability to modify when heat release occurs. Optical images of these three sample conditions are provided in Figure S2. For this work, two parameters were defined to understand the influence of defects on the resulting decomposition behavior: (1) relative defect density per unit volume (ρ_(*hkl*)_) and (2) average number of defects per crystal (σ_(*hkl*)_).

**Table 1:** Coherence Length, Relative Defect Density, and Average Defects Per Crystal for Recrystallized AP Samples Drop-Casted at Various Temperatures

concentration [mg AP: mL MeOH: mL EtOAc]	temp [°C]	preferential crystallographic orientation	CL_(*hkl*)_ [μm]	relative defect density [μm^–3^]	average defects per crystal [#]
15:1:1	20	(210)	0.0154	2.74 × 10^5^	1.19 × 10^9^
30:1:1	40	(002)	0.0136	4.00 × 10^5^	4.19 × 10^8^
30:2:0	80	(002)	0.0114	6.78 × 10^5^	1.56 × 10^9^

For calculating the relative defect density per unit volume, ρ_(*hkl*)_, the coherence length is used because it is a volume-averaged measurement over the entire thin film. The coherence length measurement represents the average length in the crystal before a defect occurs in the crystal lattice. Because this measurement is volume-averaged, it is considered to be independent of crystal size. For the drop-casted samples, where we assume isotropic defect distribution, we defined a relative defect density (ρ_(*hkl*)_) in the three-dimensional crystal as ρ_(*hkl*)_ = [CL_(*hkl*)_]^−3^, where CL_(*hkl*)_ is the coherence length with respect to the preferential crystallographic plane. The resulting value gives a relative number of defects per unit volume.

**Figure 5:**
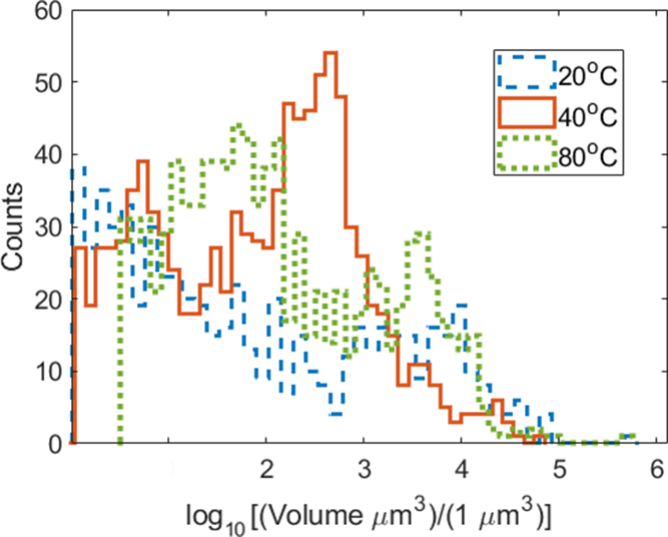
Histogram of log of particle volume distribution for recrystallization parameters (a) 15:1:1, 20 *°*C, 0 mm/s; (b) 30:1:1 40 *°*C, 0 mm/s; and (c) 30:2:0, 80 *°*C, 0 mm/s.

For calculating the average number of defects per crystal, σ_(*hkl*)_, the following equation was utilized: σ_(*hkl*)_ = *V* * [CL_(*hkl*)_]^−3^, where V is the average volume of crystals produced at the processing condition. Here, the average volume of the crystals produced under each processing condition is measured using CT measurements. The logarithmic (for clarity) volume distribution of at least 490 particles for each of the three processing conditions chosen is shown in Figure 5. Volumes that were smaller than 3 μm^3^ were excluded since these detected objects are below the resolution (i.e., less than 3 voxels on a side). Logarithm of particle volumes indicates a bimodal distribution. The smaller mode shifts to smaller sizes for a lower processing temperature. The larger mode was greatest for the dropcast samples made in the 20 °C case. This result is expected since the evaporation rate is slower at lower temperatures, and thus, crystal growth should be more dominant than nucleation compared to the other conditions. Interestingly, the 80 °C large mode was located at a larger volume than the 40 °C condition. The samples processed at 20, 40, and 80 °C had an average particle volume of 4360, 1050, and 2310 μm^3^, respectively (significant figures were based on raw volume data).

Based on our results and calculations, the data are presented in Table 1. Relative defect density increases as crystallization temperature increases, an expected result as increasing rates of supersaturation typically do not allow sufficient time for monomers to orient and attach in a perfect, coherent structure.^35,36^ Interestingly, the average defects per crystal also increased in the crystals dropcast in MeOH without antisolvent (EtOAc) and at 80 °C temperature. As the 80 °C temperature used is higher than the boiling point of MeOH, it is possible that the higher temperature and faster evaporation rate both caused the increased defect formation per crystal, even though the average crystal size was larger for the samples crystallized at 80 °C compared to the 40 °C samples.

**Figure 6:**
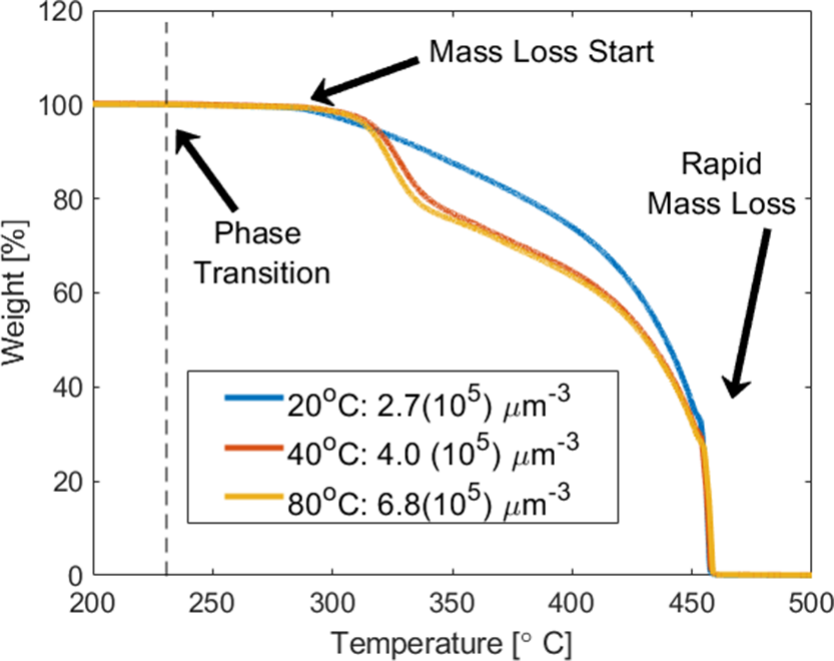
TGA plot for recrystallized samples processed at 15:1:1, 20 °C, 0 mm/s; 30:1:1, 40 °C, 0 mm/s; and 30:2:0, 80 °C, 0 mm/s. Defect density is listed with each sample condition for ease of comparison.

Next, the recrystallized sample mass loss kinetics were measured as a function of temperature (Figure 6). All recrystallized samples exhibited mass loss starting at temperatures that ranged from 288 °C (20 °C dropcast sample) to 295 °C (40 and 80 °C dropcast sample case). This mass loss behavior occurred after the orthorhombic to cubic phase transition near 240 °C.^37^ This observation suggests that defects present in recrystallized, orthorhombic AP impacted the transition to the cubic phase. This aspect likely altered the cubic phase microstructure, which affected the subsequent decomposition process. Even though the start of decomposition occurs at similar temperatures for the samples with increased defects, the overall qualitative behavior is different at higher temperatures. Near 320 °C, the greater defect density samples lose mass quicker, thus changing the decomposition kinetics.

**Figure 7:**
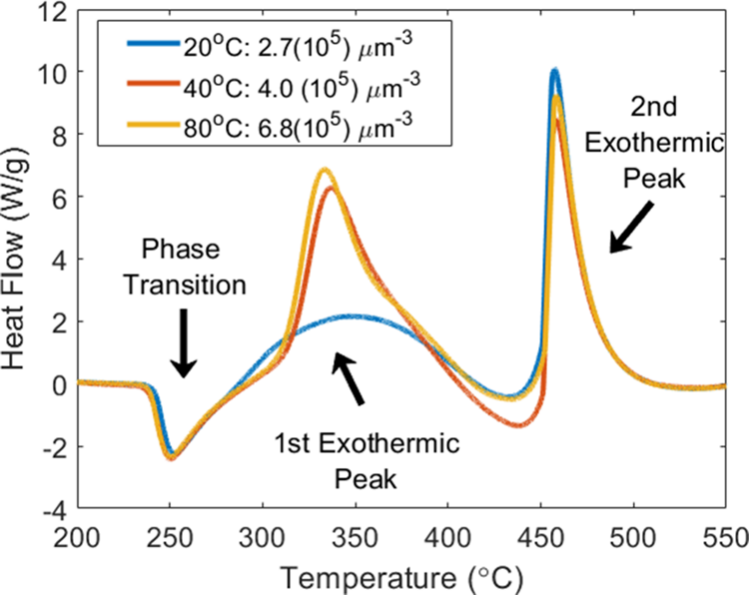
DSC plot for recrystallized samples processed at 15:1:1, 20 °C, 0 mm/s; 30:1:1, 40 °C, 0 mm/s; and 30:2:0, 80 °C, 0 mm/s (dropcast conditions).

**Figure 8:**
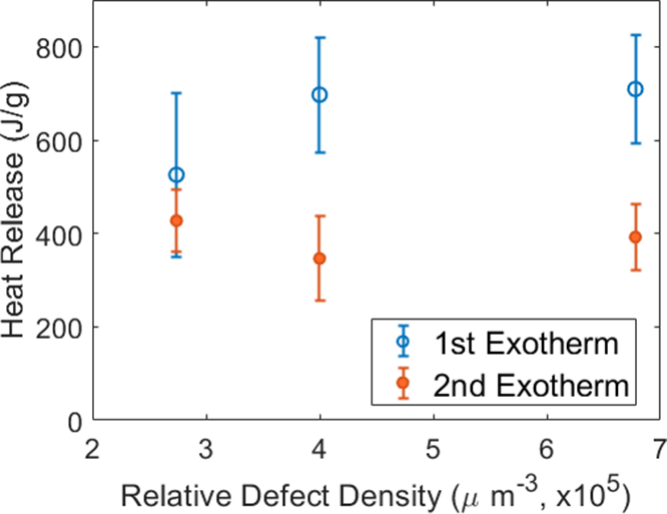
Enthalpy plot for the first and second exothermic peaks from DSC analysis for samples processed at 20, 40, and 80 °C.

As the change in mass loss should also correspond to a change in the heat release dynamics, we studied the dynamic thermal release of the AP using DSC. Figure 7 displays the DSC results of the recrystallized samples. Data from all samples consist of the endothermic orthorhombic to cubic phase transition and two exothermic peaks (the first exothermic peak may be the convolution of multiple events, since a slight shoulder appears toward the end of the event). The samples drop-casted at (15:1:1, 20 °C), (30:1:1, 40 °C), and (30:2:0, 80 °C) conditions, respectively. The second exothermic peak temperature locations were 456, 458, and 458 °C, respectively. The areas under the exothermic peaks represent energy release from the system. These values are presented in Figure 8 where all samples released the same, within uncertainty, total amount of energy. Interestingly, the first exothermic reaction increased with relative defect density, whereas the second exothermic peak decreased with the same conditions, which aligns with the mass loss behavior. Increased defect density shifted the energy release of the system to lower temperatures above a certain defect density. A similar trend was observed as a function of defects per crystal instead of per unit volume.

## Discussion

These observations indicate that the processing conditions for AP crystallization influence the decomposition kinetics. The blade speed, solvent composition, and temperature are processing parameters that affect the rate of evaporation of the solvent during the recrystallization process. It was found that the coherence length decreased with increased blade speed. The coherence length was hypothesized to have decreased due to increased evaporation rates and supersaturation rates that led to faster nucleation rates, resulting in less ordered crystallinity.^38^ A less ordered crystal could explain why the relative defect density for the crystal microstructure also increased.

The process by which AP lattice structure imperfections affect low-temperature decomposition (>350 °C)^39,40^ is evident by the shift to increased heat release of the first exothermic peak. The defect structure plays an important role in the decomposition of AP because the defects act as initiation reaction sites.^39,41,42^ As such, we believe that the relative defect density directly influences AP thermal decomposition behavior. The change in the relative defect density is likely to increase charge transport rates in AP because the imperfect lattice allows for proton migration through the lattice structure. Studies have shown that low-temperature reaction occurs in intermosaic grain boundaries,^40^ so increased relative defect density could increase the rate of proton transportation and conductivity of AP. The observed decomposition kinetics are likely due to lattice changes enabling an increase in proton exchange rate between the ammonium and perchlorate ions.

Interestingly, the initial defects present in the crystals were measured from XRD at room temperature where AP is in the orthorhombic phase, while all of the heat release and mass loss occur after the cubic phase transition. This aspect suggests that initial crystal microstructures created and measured in the orthorhombic phase will influence where the cubic phase nucleates. The initial mass loss occurs at temperatures slightly lower than the temperature of the heat release. Therefore, the bulk of the exothermic event is likely the oxidation of ammonia and perchloric acid in the gas phase and not condensed phase reactions. Subsequent reactions of gaseous intermediate species will release heat and decrease the sample mass as gas forms from the heat feedback to the crystals.

These results are important for two reasons. First, these findings show that the amount of heat released from the low-temperature decomposition regime is altered by controlling the recrystallization process via charge transport from the defects. Keenan and Seigmund^39^ summarized the early observations between AP treatment history and defects on the low-temperature decomposition. The current results indicate that engineered defects within the crystal will enable the adjustment of the crystallization process to alter decomposition rates. The second reason is that more detailed characterization is necessary for those studies recrystallizing AP with additives used as burning rate modifiers. The increased defect density increases charge transfer within the crystal, but additives such as metal oxides, graphene-based materials, etc., are also believed to affect those processes in addition to catalytic or thermodynamic effects.^4,9,43^ Therefore, additional attention must be given to changes to the AP microstructure as well as any additive effects. In these formulations, a more comprehensive study is needed to quantify the dominant mechanism involved during decomposition particularly as heating rates increase to those relevant to combustion (∼10^6^ K/s).

## Conclusions

In this work, a MGC was used to control the crystallization of orthorhombic AP and understand the relationship between crystal properties (e.g., morphology, size, defects) and heat release behavior. A large parameter space was explored to determine the variety of crystal properties achievable through MGC. Dropcast and slow blade speeds produced larger, more isotropic crystals compared to faster blade speeds, where a nucleation-dominated regime results in the formation of feather-like crystal morphologies. Both the particle size and film thickness decrease as the blade speed increases. Particle size ranged from 18 to 110 μm, and film thickness ranged from 200 nm to 14 μm. The size effects are coupled with changes in orientation where films less than 0.5 μm thick exhibit preferential orientation associated with higher energy surfaces (e.g., (200), (011)). Varying the crystallization process also resulted in different values for defect density within the AP crystals, where lower crystallization temperatures resulted in lower average defect density and higher crystallization temperatures resulted in higher average defect density. DSC and TGA results show a correlation between defect density and the heat release profile. An increase in defect density shifted the heat release from the high- to low-temperature decomposition regime. It is believed that changes to orthorhombic AP affect the defect density during the orthorhombic to cubic phase transition before decomposition starts. Charge transport within the lattice will increase with additional defects that enable the decomposition to shift to lower temperatures. These findings indicate the ability to tune the energy release profile for crystalline energetic materials.

## Supporting Information Available

MGC setup and polarized optical images of samples made within the parameter space chosen, optical images and SEM images of three representative morphology regimes observed within the larger parameter space, optical images of the dropcast thin films selected for decomposition studies, reciprocal space peak location and corresponding crystallographic plane associated with ammonium perchlorate, summary of heat release values for each exothermic peak, and uncertainty listed (PDF)

## References

[ref1] Bennett A. J.; Foroughi L. M.; Matzger A. J. Perchlorate-Free Energetic Oxidizers Enabled by Ionic Cocrystallization. J. Am. Chem. Soc. 2024, 146 (3), 1771–1775. 10.1021/jacs.3c12023. 38181408

[ref2] Zhao P.; et al. A green oxidizer based on 1,2,3-triazole with a high oxygen balance of + 23.3%: a promising replacement of ammonium perchlorate in solid propellants. Journal of Materials Chemistry A 2024, 12 (23), 13682–13688. 10.1039/D4TA01653J.

[ref3] Abdelaziz A.; et al. Application of co-crystallization method for the production of ammonium perchlorate/ammonium nitrate oxidizer for solid rocket propellants. Chemical Engineering Journal 2024, 487, 150654 10.1016/j.cej.2024.150654.

[ref4] Fehlberg S.; et al. Decomposition of Ammonium-Perchlorate-Encapsulated Nanoscale and Micron-Scale Catalyst Particles. Journal of Propulsion and Power 2020, 36 (6), 862–868. 10.2514/1.B37923.

[ref5] Sutton G. P.; Biblarz O. Rocket Propulsion Elements; John Willey & Sons. Inc.: New York, 2001.

[ref6] Gross M. L.; Beckstead M. W. Steady-state combustion mechanisms of ammonium perchlorate composite propellants. Journal of propulsion and power 2011, 27 (5), 1064–1078. 10.2514/1.B34053.

[ref7] Dennis C.; Bojko B. On the combustion of heterogeneous AP/HTPB composite propellants: A review. Fuel 2019, 254, 115646 10.1016/j.fuel.2019.115646.

[ref8] Kuo K.K.-Y.; Acharya R. Applications of turbulent and multiphase combustion; John Wiley & Sons, 2012.

[ref9] Kalman J. Are all solid propellant burning rate modifiers catalysts?. Propell. Explos. Pyrotech. 2022, 47 (9), e202200148 10.1002/prep.202200148.

[ref10] Chae H. K.; et al. A route to high surface area, porosity and inclusion of large molecules in crystals. Nature 2004, 427 (6974), 523–527. 10.1038/nature02311. 14765190

[ref11] Boldyrev V. Thermal decomposition of ammonium perchlorate. Thermochimica acta 2006, 443 (1), 1–36. 10.1016/j.tca.2005.11.038.

[ref12] Jacobs P. W. M.; Whitehead H. Decomposition and combustion of ammonium perchlorate. Chemical Reviews 1969, 69 (4), 551–590. 10.1021/cr60260a005.

[ref13] Patel J.; et al. Initiation Step in the Condensed Phase Decomposition Process of Ammonium Perchlorate. ChemistrySelect 2023, 8 (46), e202303409 10.1002/slct.202303409.

[ref14] Tolmachoff E. D.; et al. Effects of select metal oxides on the onset, rate and extent of low temperature ammonium perchlorate decomposition. Journal of Energetic Materials 2022, 40 (4), 429–444. 10.1080/07370652.2021.1895914.

[ref15] Feltenberger H. H.; Kalman J.&nbsp;The Effects of Simple Copper Containing Particles on the Thermal Decomposition of Ammonium Perchlorate. In AIAA SCITECH 2023 Forum, 2023.

[ref16] Kalman J.; et al. Nano-Computed Tomographic Measurements of Partially Decomposed Ammonium Perchlorate Particles. Propellants, Explosives, Pyrotechnics 2017, 42 (9), 1111–1116. 10.1002/prep.201700079.

[ref17] Elban W.; et al. Microstructural basis for enhanced shock-induced chemistry in single crystal ammonium perchlorate. Journal of Propulsion and Power 1995, 11 (1), 24–31. 10.2514/3.23836.

[ref18] Verneker V. R. P.; Rajeshwar K. Effect of prior mechanical and thermal treatment on the thermal decomposition and sublimation of cubic ammonium perchlorate. J. Phys. Chem. Solids 1976, 37 (1), 63–66. 10.1016/0022-3697(76)90181-5.

[ref19] Yeh I.-C.; Andzelm J. W. Computational Study of Structural and Energetic Properties of Ammonium Perchlorate at Interfaces. The Journal of Physical Chemistry C 2021, 125 (22), 12297–12304. 10.1021/acs.jpcc.1c01551.

[ref20] Ramirez D.; Kalman J.; Essel J. Influence of Hydroxyl-Terminated Polybutadiene Variants on the Wettability of Ammonium Perchlorate. Journal of Propulsion and Power 2022, 38 (4), 647–655. 10.2514/1.B38607.

[ref21] Khan M. A. S.; et al. Morphology of ammonium perchlorate in the presence of ethylene glycol as an additive: a first principle study. CrystEngComm 2019, 21 (48), 7519–7527. 10.1039/C9CE01262A.

[ref22] Kohga M.; Tsuzuki H. Crystal habit modification of ammonium perchlorate by ethylene glycol. Advanced Powder Technology 2010, 21 (4), 443–447. 10.1016/j.apt.2010.01.004.

[ref23] Kohga M.; Tsuzuki H. Burning-Rate Characteristics of Composite Propellant Using Ammonium Perchlorate Modified by Ethylene Glycol. Journal of Propulsion and Power 2011, 27 (3), 668–674. 10.2514/1.49719.

[ref24] Smith N.; et al. Selective Morphological and Polymorphic Control of CL-20 Thin Films Using Meniscus-Guided Coating. Crystal Growth & Design 2022, 22 (2), 1164–1171. 10.1021/acs.cgd.1c01103.

[ref25] Guthrie S. M.; Smilgies D.-M.; Giri G. Controlling Polymorphism in Pharmaceutical Compounds Using Solution Shearing. Crystal Growth & Design 2018, 18 (2), 602–606. 10.1021/acs.cgd.7b01686.

[ref26] Giri G.; et al. High-Mobility, Aligned Crystalline Domains of TIPS-Pentacene with Metastable Polymorphs Through Lateral Confinement of Crystal Growth. Adv. Mater. 2014, 26 (3), 487–493. 10.1002/adma.201302439. 24133041

[ref27] Guthrie S. M.; et al. Probing Molecular Assembly of Small Organic Molecules during Meniscus-Guided Coating Using Experimental and Molecular Dynamics Approaches. The Journal of Physical Chemistry C 2021, 125 (11), 6269–6277. 10.1021/acs.jpcc.0c10531.

[ref28] Conley A. M.; et al. Enhancing Organic Semiconductor Molecular Packing Using Perovskite Interfaces to Improve Singlet Fission. Adv. Funct. Mater. 2023, 33 (47), 2303232 10.1002/adfm.202303232.

[ref29] Ghorbanpour A.; et al. Oriented UiO-66 thin films through solution shearing. CrystEngComm 2018, 20 (3), 294–300. 10.1039/C7CE01801K.

[ref30] Jung S.; et al. Conductive, Large-Area, and Continuous 7,7,8,8-Tetracyanoquinodimethane@HKUST-1 Thin Films Fabricated Using Solution Shearing. ACS Applied Materials & Interfaces 2021, 13 (8), 10202–10209. 10.1021/acsami.1c00640. 33605712

[ref31] Le Berre M.; Chen Y.; Baigl D. From Convective Assembly to Landau–Levich Deposition of Multilayered Phospholipid Films of Controlled Thickness. Langmuir 2009, 25 (5), 2554–2557. 10.1021/la803646e. 19437679

[ref32] Smith H. G.; Levy H. A. Neutron diffraction study of ammonium perchlorate. Acta Crystallogr. 1962, 15 (12), 1201–1204. 10.1107/S0365110X62003205.

[ref33] Momma K.; Izumi F. VESTA 3 for three-dimensional visualization of crystal, volumetric and morphology data. Journal of applied crystallography 2011, 44 (6), 1272–1276. 10.1107/S0021889811038970.

[ref34] Mustapha S.; et al. Comparative study of crystallite size using Williamson-Hall and Debye-Scherrer plots for ZnO nanoparticles. Advances in Natural Sciences: Nanoscience and Nanotechnology 2019, 10, 045013 10.1088/2043-6254/ab52f7.

[ref35] Markov I. V. Crystal Growth for Beginners; World Scientific, 2016.

[ref36] Ye Y.-H.; et al. Self-assembling three-dimensional colloidal photonic crystal structure with high crystalline quality. Appl. Phys. Lett. 2001, 78 (1), 52–54. 10.1063/1.1337619.

[ref37] Vyazovkin S.; Wight C. A. Kinetics of Thermal Decomposition of Cubic Ammonium Perchlorate. Chem. Mater. 1999, 11 (11), 3386–3393. 10.1021/cm9904382.

[ref38] Ma Z.; et al. Preparation and characterization of superfine ammonium perchlorate (AP) crystals through ceramic membrane anti-solvent crystallization. J. Cryst. Growth 2009, 311 (21), 4575–4580. 10.1016/j.jcrysgro.2009.06.008.

[ref39] Keenan A. G.; Siegmund R. F. Thermal decomposition of ammonium perchlorate. Quarterly Reviews, Chemical Society 1969, 23 (3), 430–452. 10.1039/qr9692300430.

[ref40] Galwey A. K.; Jacobs P. W. M.; Tompkins F. C. The thermal decomposition of ammonium perchlorate at low temperatures. Proc. R. Soc. Lond. Ser. A. Math. Phys. Sci. 1960, 254 (1279), 455–469. 10.1098/rspa.1960.0032.

[ref41] Wise H. Electrical conductivity of solid ammonium perchlorate. The Journal of Physical Chemistry 1967, 71 (9), 2843–2846. 10.1021/j100868a014.

[ref42] Khairetdinov E. F.; Boldyrev V. V. Charge transfer and thermal decomposition of NH4ClO4 crystals. J. Solid State Chem. 1974, 11 (1), 67–70. 10.1016/0022-4596(74)90147-9.

[ref43] Deng P.; Ren H.; Jiao Q. Enhanced the combustion performances of ammonium perchlorate-based energetic molecular perovskite using functionalized graphene. Vacuum 2019, 169, 108882 10.1016/j.vacuum.2019.108882.

