# Supplementary material for: Crystallization-Based Modification of Ammonium Perchlorate Heat Release
Source: Cryst Growth Des. 2024 Sep 6;24(18):7588–96. doi: 10.1021/acs.cgd.4c00769 (PMC11420949; doi:10.1021/acs.cgd.4c00769)
Supplement: Supplementary file 1 — cg4c00769_si_001.pdf [file cg4c00769_si_001.pdf]

Supplementary Information

Crystallization-based Modification of Ammonium Perchlorate Heat Release

Natalie Smith-Papin[a], Cynthia Do[b], Meagan Phister[a], Gaurav Giri[a,]*, and Joseph Kalman[b,]*

[a] Department of Chemical Engineering, University of Virginia, Charlottesville, Virginia, 22904, United States

[b] Department of Mechanical & Aerospace Engineering, California State University Long Beach, Long Beach, California, 90840

*Corresponding authors

Email: gg3qd@virginia.edu

Keywords: Ammonium perchlorate, energetic materials, orientation control

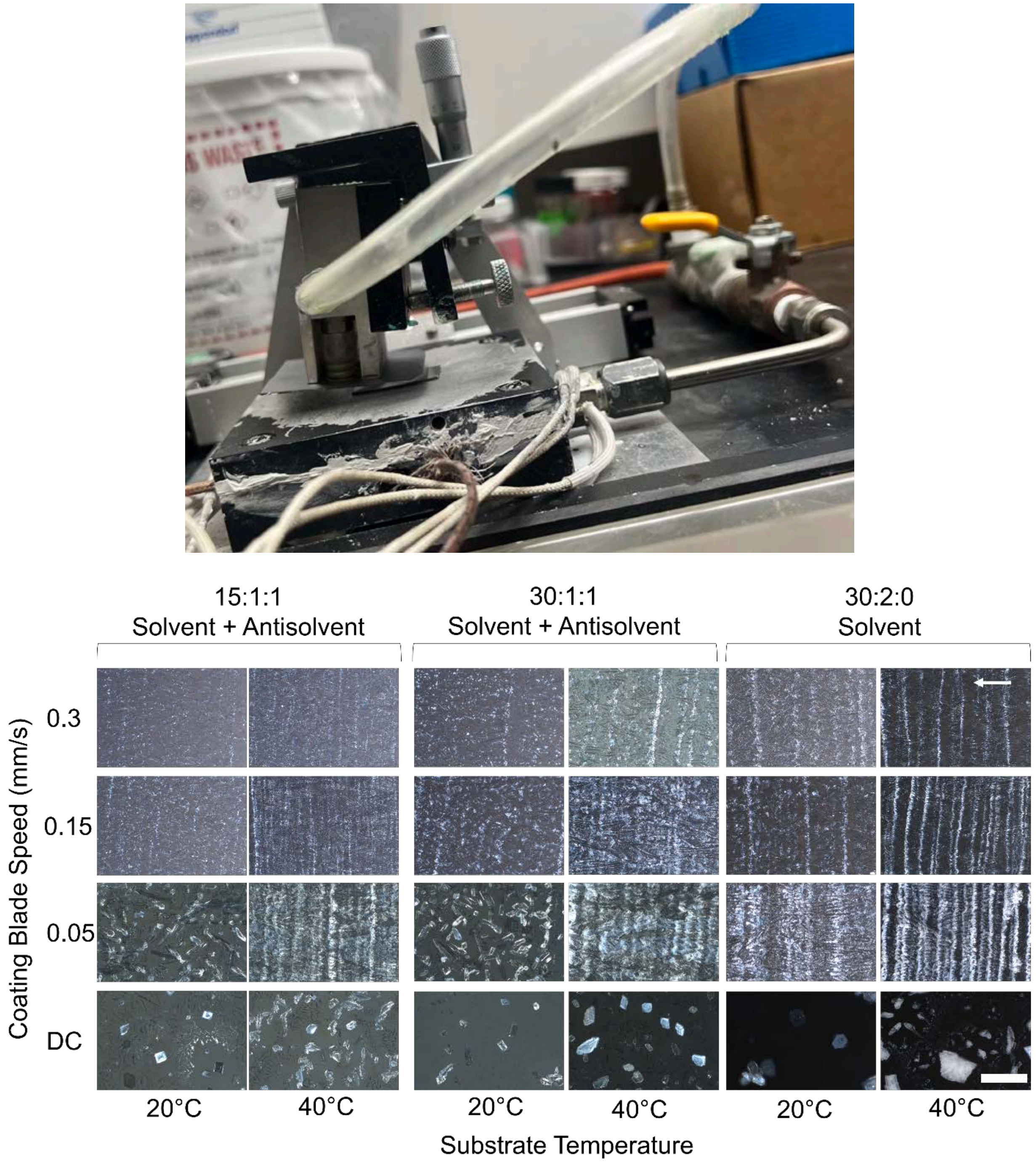

SI Figure 1a: Picture of the MGC setup. **b)** Cross-polarized optical images of all samples made within the parameter space chosen. Coating blade speed increases along the y-axis from dropcast (DC) to 0.3 mm/s, and temperature increases along the x-axis from 20 to 40 °C for three different solvent/antisolvent concentrations (15:1:1, 30:1:1, 30:2:0, where x:y:z indicates mg AP: mL MeOH: mL EtOAc). Scale bar (100 µm) in top right image is the same for all images, and arrow in top right image indicates the direction of the coating blade.

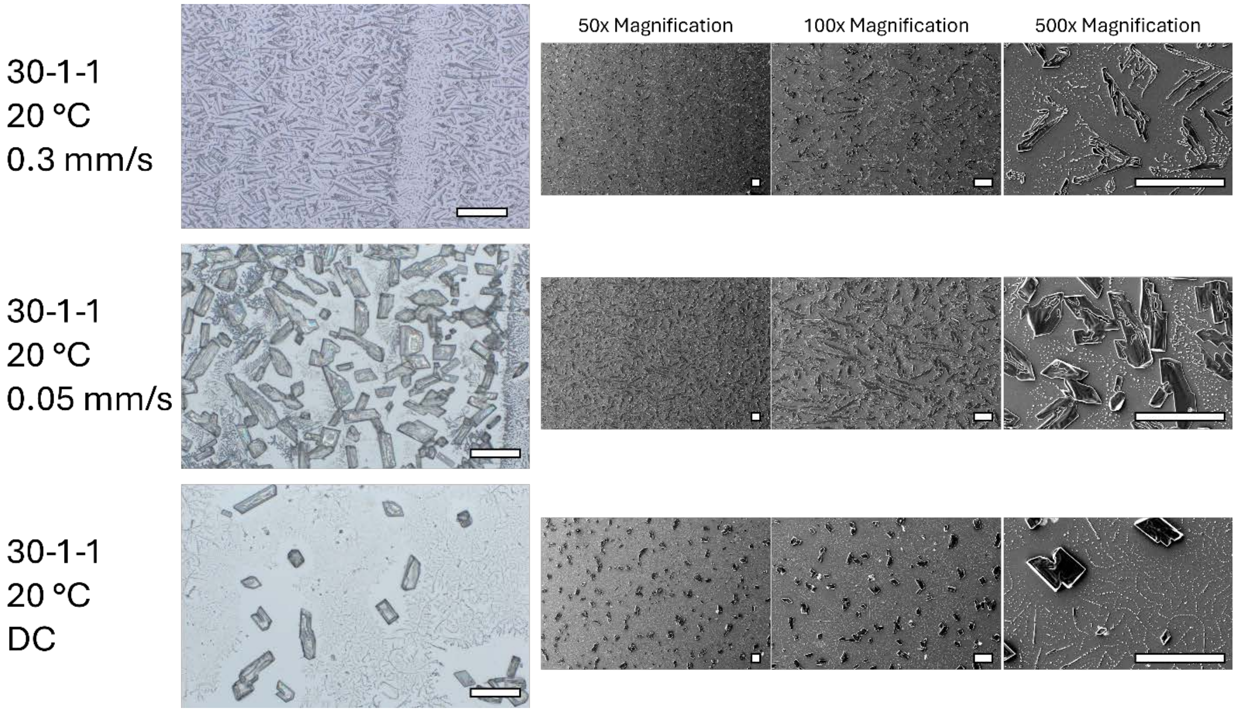

SI Figure 2: Optical microscopy (left) and scanning electron microscopy (right) images of three representative morphology regimes observed within the larger parameter space. SEM images are provided at various magnifications (10x, 100x, and 500x) to show agreement between Optical and SEM techniques. Scale bars for all images are 100 μm.

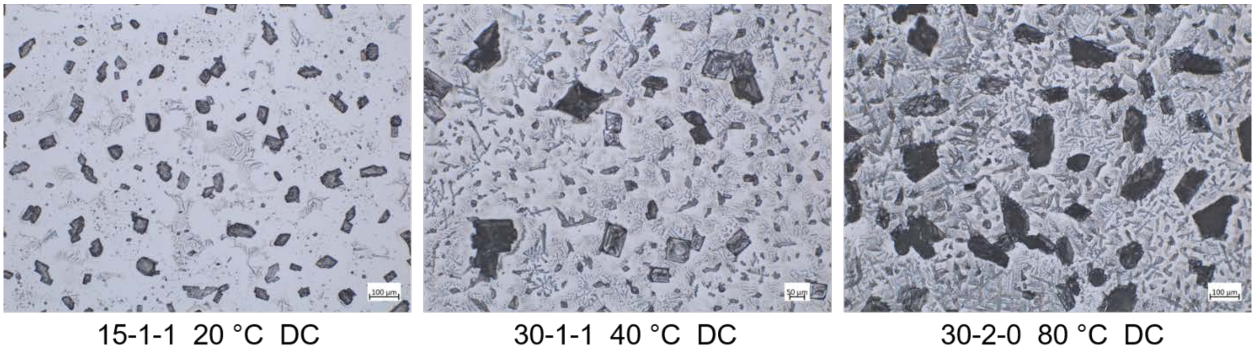

SI Figure 3: Optical images of dropcast thin films selected for decomposition studies.

**Peak location ($Å^{-1}$)**	**AP Crystallographic Plane**
**1.09**	(010)(101)
**1.37**	(200)(011)
**1.61**	(201)
**1.69**	(002)
**1.74**	(210)
**1.93**	(211)
**2.12**	(112)
**2.17**	(020)
**2.43**	(121)

SI Table 1: Reciprocal space peak location and corresponding crystallographic plane associated with ammonium perchlorate.

Relative Defect Density (μm^{-3}, x10^^{5})	1st Exotherm Heat Release (J/g)	2nd Exotherm Heat Release (J/g)
2.74	525.5±175.9	427.3±65.7
4.00	697.1±123.4	346.4±90.9
6.78	709.3±115.0	392.1±71.2

SI Table 2: Summary of heat release values for each exothermic peak. The uncertainty listed is based upon a 95% confidence interval.
